# Colorimetric Reverse Transcription Loop-Mediated Isothermal Amplification with Xylenol Orange Targeting Nucleocapsid Gene for Detection of Feline Coronavirus Infection

**DOI:** 10.3390/v17030418

**Published:** 2025-03-14

**Authors:** Kotchaporn Khumtong, Witsanu Rapichai, Wichayet Saejung, Piyamat Khamsingnok, Nianrawan Meecharoen, Siriluk Ratanabunyong, Hieu Van Dong, Supansa Tuanthap, Amonpun Rattanasrisomporn, Kiattawee Choowongkomon, Oumaporn Rungsuriyawiboon, Jatuporn Rattanasrisomporn

**Affiliations:** 1 Graduate Program in Animal Health and Biomedical Sciences, Faculty of Veterinary Medicine, Kasetsart University, Bangkok 10900, Thailand; kotchaporn.khu@ku.th (K.K.); wichayet.sa@ku.th (W.S.); 2 Department of Companion Animal Clinical Sciences, Faculty of Veterinary Medicine, Kasetsart University, Bangkok 10900, Thailand; tswitsanu@gmail.com (W.R.); piyamat.kha@ku.th (P.K.); dvhieuvet@vnua.edu.vn (H.V.D.); 3 Department of Biochemistry, Faculty of Science, Kasetsart University, Bangkok 10900, Thailand; ae.med@hotmail.com (S.R.); fsciktc@ku.ac.th (K.C.); 4 Central Laboratory (CTL), Center for Veterinary Research and Innovation, Faculty of Veterinary Medicine, Kasetsart University, Bangkok 10900, Thailand; nianrawan.mee@ku.ac.th; 5 Faculty of Veterinary Medicine, Vietnam National University of Agriculture, Hanoi 131000, Vietnam; 6 Faculty of Veterinary Medicine, Rajamangala University of Technology Tawan-ok, Bangpra, Chonburi 20110, Thailand; supansa_tu@rmutto.ac.th; 7 Interdisciplinary of Genetic Engineering and Bioinformatics, Graduate School, Kasetsart University, Bangkok 10900, Thailand; fgraapr@ku.ac.th; 8 Department of Veterinary Technology, Faculty of Veterinary Technology, Kasetsart University, Bangkok 10900, Thailand; cvtopr@ku.ac.th

**Keywords:** feline corona virus, FIP, RT-LAMP, Xylenol orange, qPCR

## Abstract

Feline infectious peritonitis (FIP), a devastating disease with near-complete mortality, is caused by the feline coronavirus (FCoV) and affects domestic cats worldwide. Herein, we report the development of a reverse transcription loop-mediated isothermal amplification (RT-LAMP) assay incorporating xylenol orange (XO) as a visual indicator for FCoV detection. The assay employed six oligonucleotide primers targeting regions of the nucleocapsid (N) gene. Under optimized conditions (65 °C, 60 min), amplification products were detected through pH-dependent colour changes in the XO dye. The RT-LAMP-XO assay exhibited high specificity for FCoV, with no cross-reactivity against other common feline viral pathogens. While the detection limit (1.7 × 10^1^ copies/µL) was an order of magnitude higher than that of qPCR, the method offered advantages in simplicity and speed compared to existing diagnostic approaches. Although less sensitive than qPCR, the RT-LAMP-XO assay may serve as a rapid screening tool when used in combination with additional primer sets. These findings demonstrate the potential utility of XO-based RT-LAMP as a simple, visual detection method for FCoV infection.

## 1. Introduction

**Figure 1:**
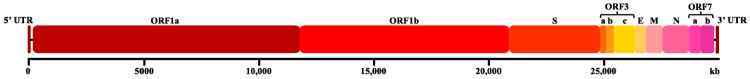
Schematic diagrams of the FCoV genome comprising the 5’ untranslated region (5’ UTR), open reading frames ORF1a/1b, spike (S) gene, ORF3abc, envelope (E), membrane (M), nucleocapsid (N), ORF7ab, and 3’ untranslated region (3’ UTR).

Feline infectious peritonitis (FIP) is a lethal immunopathological disease triggered by feline coronavirus (FCoV), an enveloped, positive-sense RNA virus with a genome of 26–32 kb [1,2]. This member of the family Coronaviridae (order Nidovirales) exhibits spherical virions measuring 120–160 nm in diameter [3,4]. The FCoV genome exhibits a linear, non-segmented organization comprising both structural and non-structural protein-coding regions [5]. The 5′ terminus contains the replicase gene complex, which consist of two overlapping open reading frames (ORF1a and ORF1b) that encode essential viral polyproteins [6]. Downstream, the genome encodes four canonical structural proteins, spike (S), envelope (E), membrane (M), and nucleocapsid (N) (Figure 1) [1]. The FCoV genome is further characterized by five accessory genes: ORF3a, ORF3b, and ORF3c, which are located in the intergenic region between S and E genes, and ORF7a and ORF7b, which are located downstream of the *N* gene [2,7]. The coronavirus spike (S) protein defines the virus’s cellular tropism and tissue-based dissemination by acting as a docking ligand for the cell receptor. Through in vitro recombination studies and the creation of viral chimeras to map areas of the S protein that might be responsible for these variations in cell tropism, cellular tropism has been examined in FIPV. A recombination event between feline coronavirus and canine enteric coronavirus produced a chimeric FCoV that encoded the canine coronavirus spike gene, which is the evolutionary outcome of serotype II FIPV [8,9,10,11,12]. The immunodominant nature of the N protein has established it as a prime target for both diagnostic development and vaccine strategies, with recent studies demonstrating the efficacy of N protein-based recombinant adenovirus vaccines in generating robust IgG and SIgA responses in feline subjects [13]. Despite its immunological significance, comparative genomic analyses have revealed that the *N* gene sequence remains notably conserved during the transition from feline enteric coronavirus (FECV) to the more virulent FIPV phenotype [14]. However, strain-specific variations in the N protein may contribute to the heterogeneous antigenic profiles observed in FIP lesions [15], suggesting that while the *N* gene is crucial for viral biology, the pathogenic mechanisms underlying FIP likely involve complex interactions between multiple viral components and host immune responses.

Diagnosing FIP before death remains difficult, particularly in the absence of body cavity effusions. The current gold standard for FIP diagnosis involves immunostaining FCoV antigens in macrophages within tissue lesions, requiring invasive tissue collection [2,16]. In FIP-affected cats, FCoV can be detected by RT-PCR in over 80% of cell-free body cavity effusions, whereas serum or blood samples frequently test negative. Both immunostaining and RT-PCR necessitate sending samples to specialized laboratories, causing delays in diagnostic results. Consequently, this can lead to unnecessary testing for other diseases, withholding necessary treatment for other treatable conditions, or delayed euthanasia in severely affected cats with FIP [17,18]. Therefore, a rapid and straightforward point-of-care test can significantly enhance the diagnostic process. Loop-mediated isothermal amplification (LAMP) is a highly efficient and specific nucleic acid amplification technique developed by Notomi et al. (2000) [19] that operates at a constant temperature, eliminating the need for thermal cycling in traditional PCR methods [20]. This method utilizes four to six primers targeting six to eight distinct regions of the target gene, significantly enhancing specificity and amplification efficiency [21]. LAMP has broad applications, including point-of-care testing, genetic testing in resource-limited settings, and the rapid detection of pathogens in clinical and environmental samples. Its simplicity, rapidity, and cost-effectiveness make it a valuable tool for diagnostic purposes, especially in settings where access to sophisticated laboratory equipment is limited [21,22,23]. Recent advancements have further improved the LAMP detection capabilities, such as real-time fluorescence and colorimetric detection, broadening its utility in various fields [21,24,25]. A previous study developed a colorimetric RT-LAMP assay for detecting FCoV, which offers simplicity and ease of interpretation and has some disadvantages. The pH sensitivity of neutral red can lead to false positives or negatives owing to environmental factors. Additionally, the dye may not be compatible with all sample types [26], potentially causing variability in results. Precipitation of the dye into visible crystals can also occur, leading to inaccurate readings [26]. The use of xylenol orange (XO) as a pH indicator is an effective method for detecting Escherichia coli DNA. XO, a cost-effective pH indicator, transitions from violet to yellow at pH levels below 6.7, allowing naked-eye observation of LAMP reaction progress. During LAMP amplification, substantial quantities of Mg_2_P_2_O_7_ and protons (H^+^) are produced, causing a significant pH drop from the initial alkaline levels (8.5–9.0) to the final acidic values (approximately 6.0–6.5) in low-buffer or non-buffered solutions. The presence of target DNA in test samples containing XO induces a colour shift from purple to yellow, indicating a positive result, whereas the absence of target DNA maintains the original violet hue, indicating a negative result. These changes are easily observable by the naked eye, facilitating rapid and straightforward diagnostics [27].

This study describes a novel colorimetric RT-LAMP technique that uses XO (RT-LAMP-XO) to detect FCoV. This involves designing specific LAMP primers based on the *N* gene. This innovative method has not been reported in previous studies on FCoV detection. This technique shows potential for clinical diagnostics with high sensitivity, specificity, and cost-effectiveness compared to current methods, enhancing FCoV detection and improving diagnostic accuracy and efficiency.

## 2. Materials and Methods

### 2.1. Ethics Statement

The study received ethical approval from the Institutional Animal Care and Use Committee of Kasetsart University, Bangkok, Thailand (Protocol code ACKU65-VET-082) and was carried out in compliance with the Declaration of Helsinki guidelines. Furthermore, sample collection was conducted with the consent of the cat’s owner.

### 2.2. Sample Collection and Sample Preparation

Pleural and/or peritoneal effusion samples (n = 77) from the thoracic and/or abdominal cavities of cats with signs of indicative FIP were collected. We obtained them from those that met at least four criteria following such consistent FIP: serum biochemical and hematological profile, abnormal clinical signs with abdominal enlargement, radiography or ultrasonography showing effusion fluid accumulating in thoracic and/or abdominal cavity, positive Rivalta’s test, or positive PCR result (FCoV detection). All samples were vortexed, diluted with 300 µL 1X sterile phosphate-buffered saline pH 8.3 at ratio of 1:3 and centrifuged at 14,000 rpm for 10 min. The supernatant was then subjected to viral RNA extraction.

### 2.3. RNA Extraction and cDNA Synthesis

Viral RNA was extracted using Viral RNA Extraction Kit according to the manufacturer’s instructions (E.Z.N.A. Viral RNA Kit; Omega Bio-tek, Norcross, GA, USA). Complementary DNA (cDNA) were synthesized using RevertAid First Strand cDNA Synthesis Kit (Thermo Scientific Inc., Waltham, MA, USA) according to the manufacturer’s instructions and used in PCR targeting *N* gene.

### 2.4. Standard Plasmid Construction

An FCoV-*N* genes amplification was performed using F9N and R9N primers as previously report [28]. An amplicon of about 1087 bp were then detected by electrophoresis and gel-purified using GeneJET Gel Extraction Kit (Thermo Scientific Inc., MA, USA). In order to clone DNA fragment of *N* gene, it was cloned into the pGEM-T vector according to pGEM^®^-T vector systems protocol (Promega, Madison, WI, USA), yielding recombinant plasmid pGEMT-*N*, which was transformed into *E*. *coli* DH5α. Positive clones with expected band 1087 bp were confirmed by colony PCR, and used as a standard plasmid throughout the experiment.

### 2.5. LAMP Primer Design

**Figure 2:**
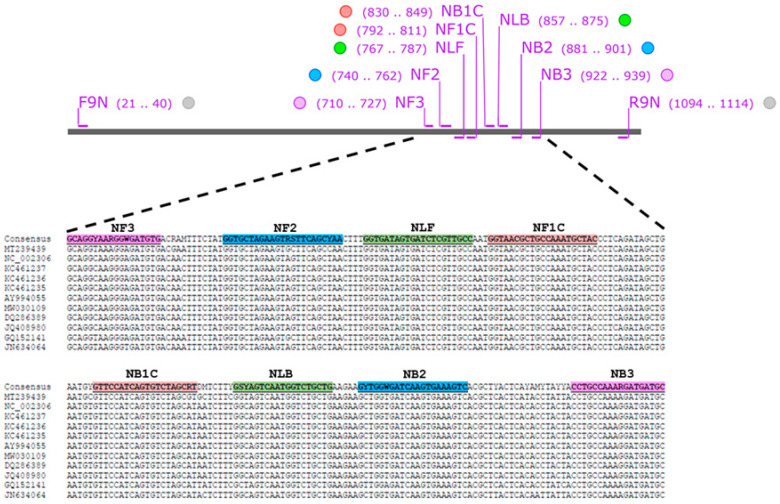
FCoV nucleotide sequences used for designing LAMP primer. Purple stripes indicate the position of NF3/NB3 primers. Blue stripes indicate the position of NF2/NB2 primers. Green stripes indicate NLF/NLB primers. Orange stripes indicate NF1C/NB1C primers.

The nucleocapsid (*N*) gene sequences of several FCoV strains with accession numbers of AY994055, DQ286389, GQ152141, JN634064, JQ408980, KC461235, KC461236, KC461237, MT239439, MW030109, and NC_002306 were obtained from GenBank database (https://www.ncbi.nlm.nih.gov/nucleotide/ (accessed on 19 June 2024). They were performed multiple sequence alignment using MEGA X software (https://megasoftware.net/) and the consensus sequence was created by BioEdit version 7.2 software (https://bioedit.software.informer.com/7.2/ (accessed on 19 June 2024)). LAMP primer set was designed using Primer Explorer V5 software (http://primerexplorer.jp/lampv5e/index.html (accessed on 19 June 2024). A set of six oligonucleotide primers targeting 230 bp of FCoV *N* gene comprising two outer (NF3 and NB3), two inner (NFIP and NBIP), and two loop primers (NLF and NLB). The information for the primer sets used in this study is presented in Figure 2 and listed in Table 1.

### 2.6. RT-LAMP Reaction and Optimization

An RT-LAMP with xylenol orange (RT-LAMP-XO) reaction was prepared in a total volume of 15 µL. The master mix contained 1.5 µL of 10X ammonium sulphate buffer pH 8.5 (100 mM (NH_4_)_2_SO_4_, 500 mM KCl, 20 mM MgSO_4_, and 1% *v*/*v* Tween-20), 3.6 µL of 25 mM MgCl_2_, 2.1 µL of 10 mM dNTPs (Thermo Scientific, Wilmington, NC, USA), 1.5 µL of 10X LAMP primer mix (0.2 µM of each outer primers, 1.6 µM of each FIP, BIP and 0.4 µM of each LF, LB), 0.6 µL of 2.5 mM xylenol orange (XO) (Sigma-Aldrich, St. Louis, MO, USA), 0.6 µL of 8 U/μL *Bst* 2.0 Warmstart DNA polymerase (New England BioLabs, Ipswich, MA, USA), 0.3 µL 200 U/µL RevertAid reverse transcriptase (Thermo Scientific, Wilmington, NC, USA), 3.8 µL of RNase-free water (Apsalagen, Bangkok, Thailand), and 1 µL of 10 ng RNA sample. The reactions were incubated at 65 °C for 60 min and then inactivated at 80 °C for 5 min using a thermal cycler (T100; Bio-Rad, Hercules, CA, USA). Reaction with nuclease-free water instead of RNA was used as negative control. The RT-LAMP-XO master mixes were prepared in a PCR cabinet to avoid contamination. After the reaction was stopped, it was visualized with the naked eye, and a positive reaction demonstrated a yellowish colour, whereas a negative reaction demonstrated a violet colour. For optimization of the RT-LAMP-XO assay, five factors of temperatures (ranging from 61 °C to 70 °C), times (10, 20, 30, 40, 50, and 60 min), MgCl_2_ concentrations (2, 4, 6, 8, and 10 mM), dNTP concentrations (0.6, 1.0, 1.4, 1.8, and 2.2 mM), and *Bst* DNA/RNA polymerase (0.08, 0.16, 0.24, and 3.32 U/μL) were determined.

### 2.7. Analytical Specificity of RT-LAMP-XO

The specificity of the RT-LAMP-XO colorimetric assay was examined under optimal conditions using viral DNA and RNA extracted from clinical samples infected with other feline viruses, including feline calicivirus (FCV), feline leukemia virus (FeLV), feline herpesvirus (FHV), feline immunodeficiency virus (FIV), feline panleukopenia virus (FPLV), Crandell-Rees Feline Kidney (CRFK) cells, and whole blood cells. The recombinant plasmids pGEMT-*N* and RNase-free water were used as positive and negative controls. RT-LAMP amplification was detected using a CFX96 Touch Real-Time PCR Detection System (Bio-Rad, Hercules, CA, USA) and colorimetric detection was performed with the naked eye.

### 2.8. Quantitative PCR (qPCR) Reaction Condition

For comparison of the sensitivity of RT-LAMP for FCoV detection, the qPCR master mix reaction of 25 µL contains 12.5 µL of 2X Maxima SYBR Green qPCR Master Mix (Thermo Scientific, Wilmington, NC, USA), 0.6 µL each of 10 µM NF2 and NB2 primer, 1 µL recombinant plasmid pGEMT-*N* template, and 10.3 µL RNase-free water. The qPCR conditions were 40 cycles of denaturation at 94 °C for 30 s, annealing at 56 °C for 30 s, and extension at 72 °C for 1.5 min, followed by a final extension at 72 °C for 5 min. The amplification products were monitored at each elongation step using a CFX96 Touch Real-Time PCR Detection System (Bio-Rad, Hercules, CA, USA).

### 2.9. Comparative Sensitivity of RT-LAMP-XO and qPCR Assay

Ten-fold serial dilutions (10^6^ to 10^0^ copies/µL) of the recombinant plasmid pGEMT-*N* were prepared and each dilution was used as templates for both RT-LAMP-XO assays and qPCR to determine the limit of detection (LOD). The LOD of the methods was defined as lowest copy number detected by colorimetric detection with the naked eye and fluorescent signals. The following equation was used to calculate the concentration of the recombinant plasmid pGEMT-*N* DNA template in terms of copy number per microliter [29]:

(1) $$\mathrm{DNA}\;\mathrm{copy}\;\mathrm{number}(\mathrm{copies}/\mu L)=\frac{X(\mathrm{ng}/\mu L)\times K_{A}}{Y(\mathrm{bp})\times 10^{-9}\times 660}$$

where X is the concentration of recombinant plasmid, Y is the number of base pairs in the vector, and K_A_ is Avogadro’s constant ($6.022\;\text{×}\;10^{23}$).

### 2.10. Diagnosis and Statistical Analysis of FCoV Infected Clinical Sample

Sensitivity and specificity using RT-LAMP-XO and qPCR assay were calculated and compared. The data were expressed as percentages to determine the effectiveness of LAMP detection using the two-by-two table. To estimate the diagnostic parameters, including sensitivity, specificity, FCoV prevalence, positive predictive value (PPV), negative predictive value (NPV), and overall accuracy of the RT-LAMP-XO assay for FCoV detection, we utilized MedCalc’s Diagnostic Test Evaluation Calculator (available at https://www.medcalc.org/calc/diagnostic_test.php (accessed on 19 June 2024)). This analysis employed data from 77 pleural and/or peritoneal effusion samples previously diagnosed with qPCR.

## 3. Results

### 3.1. Design and Verification of FCoV LAMP Primer

**Figure 3:**
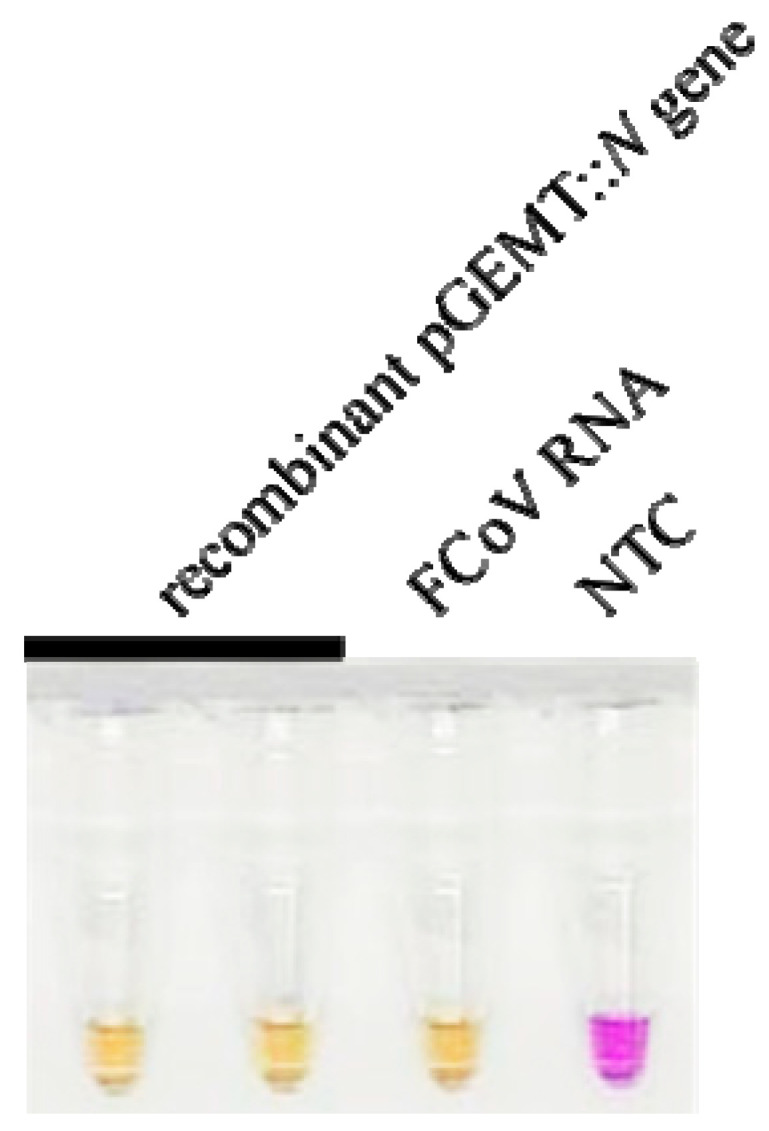
FCoV LAMP primer validation. Yellow colour shows positive reaction and violet colour shows negative reaction. NTC, negative control.

For the development of RT-LAMP with xylenol orange (RT-LAMP-XO), six primers were designed based on eight different regions of the nucleocapsid (N) gene of the FCoV/FIPV sequences. These comprising two outer primers (NF3/NB3), two inner primers (NFIP/NBIP), and two loop primers (NLF/NLB). To verify whether the designed LAMP primers functioned suitably, they were then examined with a recombinant plasmid harbouring the FCoV *N* gene and FCoV RNA at 65 °C for 60 min. After the reaction, the change in RT-LAMP-XO from violet-to-yellow confirmed that our novel LAMP primer design has the potential for further experiments (Figure 3).

### 3.2. Optimization of RT-LAMP Conditions for FCoV Detection

**Figure 4:**
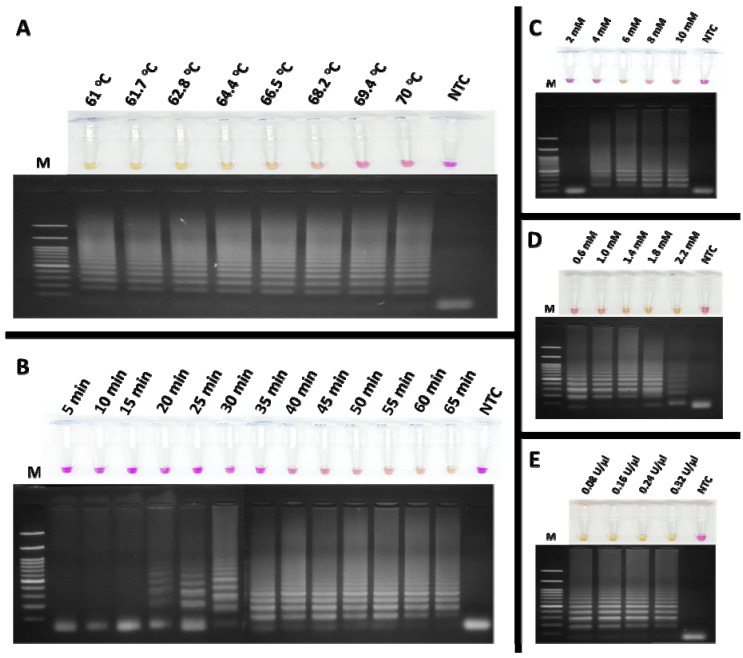
Optimization of the colorimetric RT-LAMP-XO assay for FCoV detection involved evaluating several key parameters: (**A**) the effect of temperature; (**B**) the effect of incubation time, with the optimal amplification time determined to be 60 min; (**C**) the effect of MgCl_2_ concentration, with 6 mM identified as optimal; (**D**) the effect of dNTP concentration, with 1.4 mM being optimal; and (**E**) the effect of Bst DNA/RNA polymerase activity, with 0.32 U/µL selected as the optimum enzyme activity. Lane M represents the 100 bp DNA Ladder Marker III (Yeastern Biotech, Taiwan, China), and lane NTC is the negative control using RNase-free water.

To detect feline coronavirus (FCoV) using the RT-LAMP assay, xylenol orange (XO) dye was employed as an indicator, enabling the visual detection of positive reactions by a colour change from violet to yellow. The amplification products were verified by agarose gel electrophoresis. The optimal reaction conditions were systematically determined as follows. The effect of temperature on the colorimetric RT-LAMP-XO assay was examined using a gradient range of 61–70 °C, with a fixed amplification time of 60 min. As shown in Figure 4A, reactions within this range yielded positive results except for 69.4 and 70 °C, displaying a ladder-like band pattern. Based on the manufacturer’s recommended temperature range for the *Bst* DNA/RNA polymerase (60–72 °C), 65 °C was selected as the optimal temperature for further experiments. Next, the amplification time was optimized by incubating the reactions for durations ranging from 5 to 65 min. Figure 4B demonstrates that detectable amplification clearly occurred at 60 min, as evidenced by the yellow coloration and distinct band pattern (lane 13), which was chosen as the optimal incubation time for subsequent experiments. The effect of MgCl_2_ concentration was assessed by testing concentrations of 2, 4, 6, 8, and 10 mM. As shown in Figure 4C, a clear ladder-like band pattern was observed at 6 mM (lane 3), which was selected as the optimal concentration. Similarly, dNTP concentrations ranging from 0.6 to 2.2 mM were evaluated. Figure 4D indicates that 1.4 mM provided optimal results for both colorimetric detection and band pattern clarity (lane 3). Deviations from the optimal MgCl_2_ and dNTP concentrations led to inconsistent results with clear visualization. For *Bst* DNA/RNA polymerase, activities ranging from 0.08 to 0.32 U/µL were tested. As shown in Figure 4E, 0.32 U/µL provided the best results, producing a concentrated yellow colour and a strong band pattern (lane 5). These optimized conditions were established to ensure reliability and reproducibility of the RT-LAMP-XO assay for FCoV detection. During the amplification process, the reaction solution is a critical determinant of both the efficiency and specificity of DNA amplification. This solution contains magnesium ions (Mg^2+^) and deoxynucleotide triphosphates (dNTPs), which are indispensable cofactors for DNA polymerase. Mg^2+^ is crucial for enzyme activity as it facilitates the formation of phosphodiester bonds during DNA synthesis. Meanwhile, dNTPs serve as the substrates that are incorporated into the new DNA strands. Therefore, their concentrations must be carefully optimized to ensure optimal amplification outcomes [30].

### 3.3. Specificity of RT-LAMP-XO for FCoV Detection

**Figure 5:**
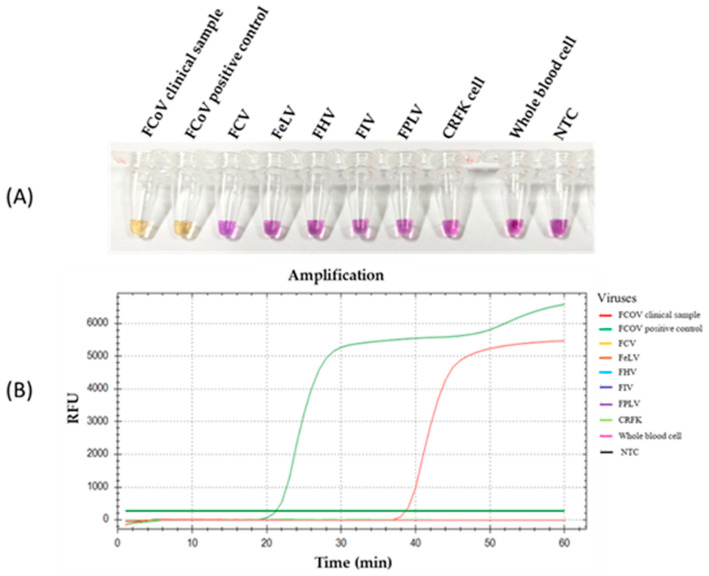
Specificity of the RT-LAMP-XO assay. (**A**) Colorimetric specificity of RT-LAMP assay using xylenol orange indicator dye. (**B**) Fluorescence signal results of the specificity analysis. FCoV, feline coronavirus; FCV, feline calicivirus; FeLV, feline leukemia virus; FIV, feline immunodeficiency virus; FPV, feline panleukopenia virus; CRFK, Crandell-Rees Feline Kidney; NTC, Negative control.

**Figure 6:**
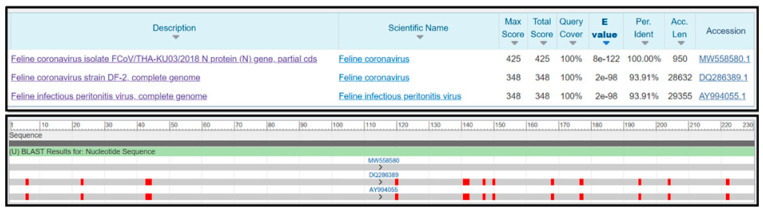
Similarity analysis of the FCoV clinical sample sequence.

The specificity of the RT-LAMP-XO reaction was evaluated by investigating potential cross-reactions with different feline viruses, including FCV, FeLV, FHV, FIV, and FPLV. The CRFK cells and whole blood cells were used as internal controls. Before the experiments, all viral genomic materials were confirmed by a veterinarian to diagnose and confirm their cause of disease. As shown in Figure 5A, the results revealed a positive reaction, with a colour change from violet to yellow observed solely in the clinical sample infected with FCoV, using the recombinant plasmid pGEMT-N as the positive control. In contrast, a negative colour change was observed in the reactions with other feline viruses. Consistently, the fluorescence detection results exhibited an amplification curve with similar trends in fluorescence signals between the FCoV clinical sample and positive recombinant plasmid control. The other tested feline viruses and internal controls did not result in any fluorescence curve above the baseline signal (Figure 5B). To confirm the identity of the positive FCoV products, Sanger sequencing was performed using the outer primers (NF3 and NB3 primers) as sequencing primers. As shown in Figure 6, the sequencing results of the FCoV clinical sample revealed 100% identity with the nucleocapsid sequence of the feline coronavirus isolate FCoV/THA-KU03/2018 (MW558580) and 93.91% identity with those of the feline coronavirus strain DF-2 (DQ286389) and feline infectious peritonitis virus (AY994055). Thus, the RT-LAMP-XO primers developed in this study exhibited remarkable specificity for detecting FCoV, without cross-reactivity with other feline viruses. To ensure the specificity of the developed LAMP primers, an in silico analysis was conducted to validate the specificity of the RT-LAMP-XO primer against other coronaviruses closely related to FCoV, such as canine coronavirus (CCoV) and porcine transmissible gastroenteritis virus (TGEV), using accession numbers KP981644 and NC_038861 as representatives of CCoV and TGEV, respectively. The in silico analysis revealed that the developed LAMP primer set for the *N* gene of FCoV exhibited high specificity for FCoV, with limited binding affinity observed for CCoV and TGEV (as shown in Figure S1 and Figure S2, respectively). This suggests that the RT-LAMP-XO assay, utilizing the developed LAMP primer set, should selectively detect FCoV without generating false-positive results due to cross-reactivity with CCoV and TGEV.

### 3.4. Comparison of RT-LAMP-XO and qPCR on Sensitivity for FCoV Detection

**Figure 7:**
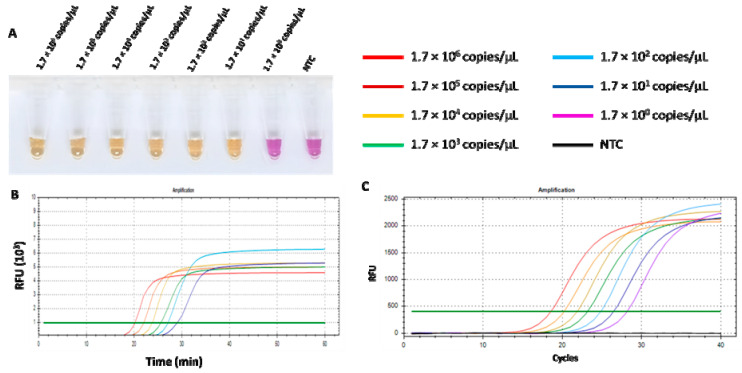
Comparison of the sensitivity of RT-LAMP-XO and qPCR for FCoV detection. (**A**) Colorimetric RT-LAMP-XO reaction. Tube a–g: RT-LAMP-XO amplicon of 10-fold serially diluted standard plasmids pGEMT-N at 1.7 × 10^6^ copies/µL to 1.7 × 10^1^ copies/µL. NTC: negative control. (**B**) Sensitivity of quantitative RT-LAMP-XO. (**C**) Sensitivity of qPCR assay.

The limit of detection (LOD) of the RT-LAMP-XO assay and qPCR was defined as the lowest copy number of recombinant pGEMT harbouring the detected *N* gene. The ten-fold dilution series was prepared and used to compare the sensitivities of both assays. As shown in Figure 7A,B, the LOD of the RT-LAMP-XO assay was 1.7 × 10^1^ copies/µL. Additionally, the RT-LAMP-XO assay results showed a clear positive colour, which was consistent with that of the fluorescent signal. While the LOD of qPCR was 1.7 copies/µL related to a cycle threshold value (Ct) of 28.18 (Figure 7C). Although the qPCR detection limit was 10 folds lower than that of RT-LAMP-XO, our proposed assay has more advantages over qPCR in its rapidity, simplicity, visualization, and convenience.

### 3.5. RT-LAMP-XO Assay Validation

A total of 77 clinical samples were subjected to FCoV detection using the same clinical samples for both RT-LAMP-XO and qPCR assays (Table S1). Table 2 presents the results of the RT-LAMP-XO compared to qPCR assay showed 17 true positives, 0 false positives, 0 false negative, and 60 true negatives. Statistical analysis of RT-LAMP-XO compared to the qPCR assay demonstrated 100% sensitivity (95% CI: 80.49% to 100%), 100% specificity (95% CI: 94.04% to 100%), 100% positive predictive value (PPV) (95% CI: 80.49% to 100%), 100% negative predictive value (NPV) (95% CI: 94.04% to 100%), 93.5% accuracy, and an FCoV prevalence of 22.08% (95% CI: 13.42% to 32.98%). These results underscore the practical value of RT-LAMP-XO for FCoV detection in clinical samples.

## 4. Discussion

The global prevalence of feline coronavirus (FCoV), a significant pathogen primarily responsible for causing feline infectious peritonitis (FIP)—a fatal disease characterized by inflammation and fluid accumulation in body cavities [26,28]—highlights the urgent need for reliable and rapid diagnostic methods [31,32,33]. Reverse transcription loop-mediated isothermal amplification (RT-LAMP) assays offer a promising solution for accessible and cost-effective FCoV diagnosis, addressing the critical requirement for timely and accurate detection in both clinical and field settings. This study presents the development of assay method for detecting FCoV using reverse transcription loop-mediated isothermal amplification (RT-LAMP). The approach employed six specifically designed primers targeting the *N* gene, which had not been previously utilized in FCoV detection assays, highlighting its potential for innovation in diagnostic techniques. Xylenol orange (XO) was used as a colorimetric indicator, facilitating visual interpretation through a distinct colour change from purple to yellow at pH 6.7, effectively simplifying result observation [27]. Diagnosing FCoV infection, linked to the often-fatal FIP, is challenging in veterinary medicine [1,34,35,36]. This study developed an RT-LAMP-XO assay for rapid, specific FCoV detection, offering a potential alternative to qPCR in resource-limited settings. The assay uses xylenol orange for easy colorimetric results [37]. However, further validation is needed to confirm its practicality as a point-of-care test (POCT) [38]. Future work will streamline RNA extraction and test the assay on minimally processed samples. Field trials in veterinary clinics will assess its user-friendliness, turnaround time, and robustness. These steps aim to make the RT-LAMP-XO assay a practical, rapid screening tool for FCoV in resource-constrained practices.

For this RT-LAMP-XO assay, optimizing parameters such as temperature, time, dNTP concentration, and Mg^2^⁺ concentration is essential for achieving maximum sensitivity and specificity in LAMP assays, especially when dealing with complex samples like clinical specimens that contain inhibitory substances. Preliminary optimization experiments can significantly reduce the time and resources needed to develop robust LAMP-based diagnostic assays [39]. The development of RT-LAMP with xylenol orange (RT-LAMP-XO) for detecting FCoV involved the design of six primers targeting eight distinct regions of the *N* gene. The primers were validated using a recombinant plasmid containing the FCoV *N* gene and FCoV RNA, with reactions conducted at 65 °C for 60 min. The successful colour change from violet to yellow in the RT-LAMP-XO assay confirmed the functionality of the primer set, demonstrating its potential for further experimental validation and application in FCoV detection. Optimizing parameters such as temperature, time, dNTP concentration, and Mg^2^⁺ concentration is essential for achieving maximum sensitivity and specificity in LAMP assays, especially when dealing with complex samples like clinical specimens that contain inhibitory substances. Preliminary optimization experiments, as demonstrated in existing studies, can significantly reduce the time and resources needed to develop robust LAMP-based diagnostic assays [19,20,21,22,23,40,41,42,43,44]. In RT-LAMP-XO reactions, temperature and incubation time are pivotal parameters that significantly impact the efficiency of nucleic acid amplification. Temperature critically affects both the activity of the *Bst* DNA polymerase and the annealing of primers to the DNA template. The optimal temperature for isothermal polymerase is approximately 65 °C [45], which is consistent with this study that uses a temperature of 65 °C for the reaction, making the enzyme’s activity optimal. Incubation time is equally crucial in LAMP reaction, determining the duration of the amplification process. Shorter incubation times may result in insufficient amplification, especially with low DNA quantities, while longer times could lead to non-specific amplification [23]. The results of determining the optimal amplification time at the optimal temperature show that the appropriate duration is 60 min, which is when the yellow colour of XO is most prominently observed. Additionally, a pivotal element in the amplification reaction is the formulation of the reaction solution, which contains two indispensable cofactors, Mg^2^⁺ and dNTPs. These components are essential for enhancing the efficiency and accuracy of the amplification process [42]. The concentration of dNTPs is crucial and must be carefully balanced with Mg^2^⁺. Optimal performance is typically achieved at around 1–1.6 mM for each dNTP, with adjustments made in conjunction with Mg^2^⁺ to prevent inhibition and ensure robust amplification. This delicate balance highlights the necessity of systematic optimization in developing effective assays [19,22,40,41]. The LAMP assay hinges critically on the presence of Mg^2+^ because *Bst* DNA polymerase, a key enzyme in the process, is Mg^2+^-dependent. Beyond that, Mg^2+^ interacts with dNTPs, primers, and templates, influencing the specificity of the LAMP reaction, the yield of amplified products, and even the formation of primer dimers. Interestingly, an excessive amount of Mg^2+^ can actually be counterproductive. Too much Mg^2+^ results in improper binding between the primer and template, which consequently reduces the specificity of the LAMP reaction [23,46,47,48]. Interestingly, exceeding Mg^2+^ concentration more the optimal level could lead to pseudo-positive results, even in negative samples. This finding underscores the importance of carefully controlling Mg^2+^ levels to ensure accurate outcomes in the amplification process [49].

This specificity is critical for diagnostic accuracy, particularly in clinical settings where co-infections or overlapping symptoms may occur [19,28]. The exceptional specificity of LAMP arises from its structural reliance on four to six primers that collectively bind to six to eight conserved regions of the target DNA. This design strategy minimizes off-target amplification. By necessitating the simultaneous hybridization of multiple primers to distinct genomic loci, LAMP ensures stringent recognition of the intended sequence, thereby reducing false-positive outcomes [19,22]. The lack of false-positive signals underscores the utility of RT-LAMP as a reliable tool for distinguishing FCoV from other feline pathogens, enhancing its potential for rapid, field-deployable diagnostics [21]. Future studies could expand this validation to include additional FCoV strains or emerging variants to further confirm primer robustness, as recommended for molecular assays targeting RNA viruses prone to genetic variability [50]. Our assay demonstrated high specificity for FCoV, as evidenced by the absence of cross-reactivity with other common feline viral pathogens. Specifically, no amplification was observed when testing with viral nucleic acid from FCV, FeLV, FHV, FIV, and FPLV. Furthermore, negative results were confirmed using CRFK feline cell lines and whole blood cell as internal controls, which ruled out non-specific binding to host DNA or background cellular components. These findings collectively validate the specificity of the six primer sets designed for FCoV detection, as they exclusively target conserved regions of the FCoV genome without cross-amplifying unrelated viral sequences. The sequencing results of the FCoV clinical sample revealed a 100% identity with the nucleocapsid sequence of the feline coronavirus isolate FCoV/THA-KU03/2018 (MW558580), indicating that the sample is genetically identical to this specific isolate [39]. Additionally, the sample showed a 93.91% identity with the nucleocapsid sequences of the feline coronavirus strain DF-2 (DQ286389) and the feline infectious peritonitis virus (AY994055) [51]. The in silico analysis conducted to validate the specificity of the RT-LAMP-XO primer set against closely related coronaviruses, such as canine coronavirus (CCoV) and porcine transmissible gastroenteritis virus (TGEV), using accession numbers KP981644 and NC_038861, demonstrated that the developed LAMP primer set for the *N* gene of FCoV exhibited high specificity for FCoV, with limited binding affinity for CCoV and TGEV. This indicates that the RT-LAMP-XO assay should selectively detect FCoV without generating false-positive results due to cross-reactivity with these related viruses. Notwithstanding the intrinsic limitations engendered by the real-world testing of clinical samples during the experimental period, the immediate empirical testing for CCoV and TGEV infections remains unfeasible. This situation underscores the critical need for future experimental validation to confirm these findings [52,53,54,55,56]. This high level of identity confirms the close genetic relationship between the clinical sample and these known strains of FCoV. The RT-LAMP-XO primers developed in this study demonstrated remarkable specificity for detecting FCoV, as they did not cross-react with other feline viruses. This specificity is crucial for accurate diagnosis and surveillance of FCoV infections, as it ensures that the primers only amplify the target FCoV sequences without detecting non-target viruses. Moreover, the sequencing results confirm the genetic identity of the clinical sample with known FCoV strains, and the RT-LAMP-XO primers provide a reliable and specific method for detecting FCoV infections in clinical settings [26,57]. The qPCR remains the gold standard for nucleic acid detection due to its high sensitivity and precise quantification, particularly in research and clinical settings requiring viral load monitoring or multiplexing [42]. However, its reliance on thermal cycling, costly equipment, and susceptibility to inhibitors in complex samples limits its utility in resource-limited environments. In contrast, LAMP operates isothermally, enabling rapid results with minimal instrumentation, making it ideal for point-of-care diagnostics [58]. Recent advancements in LAMP, such as lyophilized reagents and smartphone-based detection, have enhanced its portability and affordability, as demonstrated during the COVID-19 pandemic for field surveillance [59,60]. While LAMP exhibits slightly lower sensitivity compared to qPCR, its tolerance to inhibitors in crude samples reduces preprocessing needs, accelerating workflows [61]. The comparative evaluation of the RT-LAMP-XO assay and qPCR revealed distinct performance characteristics in terms of sensitivity. The RT-LAMP-XO assay demonstrated a LOD of 1.7 × 10^1^ copies/µL, while qPCR exhibited a 10-fold lower LOD of 1.7 × 10^0^ copies/µL. This aligns with the established literature where qPCR consistently achieves superior sensitivity due to its real-time fluorescence-based quantification and precise thermal cycling, enabling detection of ultra-low target concentrations [62]. The RT-LAMP-XO assay gave advantages in its rapidity and simplicity, as evidenced by the direct visual colorimetric readout, which eliminates the need for specialized instrumentation [26,57,63]. These features are critical for point-of-care or resource-limited settings, where speed and ease of interpretation prioritize over maximal sensitivity [64]. Furthermore, the isothermal nature of LAMP circumvents the need for thermal cyclers, reducing operational costs and technical barriers [48]. While qPCR remains indispensable for applications requiring absolute quantification, RT-LAMP-XO performance is sufficient for screening and early diagnosis, particularly in outbreaks where rapid turnaround is essential [60]. Significantly, the colour changes in the RT-LAMP-XO test match closely with fluorescent measurements, making it a reliable tool for use in the field. This is consistent with studies showing that LAMP works well with unprocessed samples and can handle substances that often interfere with qPCR tests [42,63]. The diagnostic efficacy of the LAMP-based assay was validated through a blinded evaluation of 77 clinical samples (positive and negative), demonstrating 100% sensitivity, specificity, positive predictive value (PPV), negative predictive value (NPV), and accuracy. These metrics align with stringent performance benchmarks for molecular diagnostics, as outlined by Tanner et al. (2015) in their criteria for clinical validation of LAMP assays [42,65]. Notwithstanding these strength, the study’s methodological limitations include a modest sample size, which may introduce bias or restrict generalizability. As noted, this study utilized 77 clinical samples, which may be considered relatively small, particularly given the limited number of positive samples. This limitation could potentially affect the statistical power and reliability of our findings. This concern is acknowledged, and it is agreed that larger sample sizes would significantly enhance the robustness of the method’s validation. In subsequent studies, the dataset will be expanded to include a broader and more diverse range of clinical samples. Such an expansion is expected to strengthen the statistical analysis and improve the reliability and confidence in the results. Challenges in recruiting cats during early-stage FCoV infections, when viral loads are transient or subclinical, further underscore the need for expanded validation cohorts in future studies to confirm diagnostic robustness [60]. A crucial consideration for the reliable application of the RT-LAMP-XO assay in clinical settings is the potential impact of inhibitors present in complex biological samples. Body cavity effusions, while often used for FCoV detection in FIP diagnosis, can contain a variety of substances (e.g., proteins, cellular debris, heparin) that inhibit nucleic acid amplification reactions. Such inhibitors may interfere with the activity of the *Bst* DNA polymerase or reverse transcriptase, thereby reducing amplification efficiency and potentially leading to false-negative results, especially in samples with low viral loads [66,67,68]. Diagnosing FIP before death remains difficult, particularly in the absence of body cavity effusions. To address this concern, future studies should rigorously evaluate the tolerance of the RT-LAMP-XO method to the presence of common inhibitors found in feline effusion samples, blood, and tissues [1]. This could involve spiking experiments with known inhibitors and assessing the assay’s performance. Furthermore, optimization of sample pre-processing steps, such as dilution, filtration, or the use of commercially available nucleic acid purification kits designed to remove inhibitors, is essential to minimize their effects and ensure accurate and reliable detection of FCoV. Investigating the effectiveness of different pre-processing methods and their impact on assay sensitivity will be critical for translating the RT-LAMP-XO method into a robust and dependable diagnostic tool for FCoV infection [21]. The ability of this assay to detect FCoV DNA at microgram concentrations highlights its sensitivity to low viral loads, positioning it as a promising tool for early diagnosis prior to the onset of irreversible clinical manifestations. This capability is critical for pre-emptive management of FCoV infections, where timely intervention can mitigate disease progression [1]. Furthermore, of assay, the short turnaround time and compatibility with basic laboratory equipment eliminate reliance on costly thermal cyclers, a well-documented advantage of isothermal amplification over PCR [21]. Comparative analysis with established PCR methods revealed equivalent, if not superior, sensitivity for FCoV genomic DNA detection, consistent with studies advocating LAMP’s utility in decentralized settings [69]. While the RT-LAMP-XO assay developed in this study demonstrates a simple and rapid method for FCoV detection, it is important to acknowledge its limitations regarding detection thresholds compared to qPCR. Our results showed a detection limit of 1.7 × 10^1^ copies/µL, which is an order of magnitude higher than that of qPCR. Although sample selection (pleural and/or peritoneal effusion samples) and assay optimization may have influenced this result, inherent differences in the sensitivity of the two techniques could also contribute to this discrepancy. Therefore, it is essential to recognize the possibility of false negatives, particularly in samples with low viral loads. Future studies should focus on verifying the RT-LAMP-XO assay’s performance using a wider range of clinical samples with varying viral loads, as determined by a more sensitive method like qPCR. Furthermore, modifications to the RT-LAMP-XO assay, such as incorporating techniques to enhance amplification efficiency or optimizing primer design, could potentially improve its sensitivity. In cases where high sensitivity is paramount, qPCR remains the preferred diagnostic method. However, the RT-LAMP-XO assay can serve as a valuable rapid screening tool, especially when combined with other diagnostic approaches and with careful consideration of its limitations in detecting low viral loads. Integration of colorimetric readouts enables naked-eye visualization of results, enhancing user-friendliness and enabling point-of-care deployment. This feature is particularly advantageous in resource-limited regions, where rapid, low-cost diagnostics are essential for curbing transmission and initiating targeted therapies [69,70]. Beyond diagnosis, the quantitative potential assay could facilitate monitoring therapeutic responses in FCoV-infected cats undergoing multidrug regimens, offering insights into viral load dynamics.

The RT-LAMP-XO assay offers a rapid, visual FCoV detection method with point-of-care potential. Primers targeted conserved *N* gene regions from both Type I and Type II FCoV strains. However, empirical validation against well-characterized Type I FCoV isolates, the predominant cause of natural infections, was not performed. Future studies should prioritize this validation, including comparison to qPCR, to ensure reliable Type I FCoV detection and fully evaluate clinical utility [71,72,73,74,75].

The XO-based RT-LAMP method presented in this study represents a significant step forward as a proof-of-concept assay. Despite its lower sensitivity compared to qPCR, the RT-LAMP-XO method requires RNA extraction or cDNA synthesis. Its high specificity and ease of visualization of LAMP products make it a valuable tool. This developed RT-LAMP-XO assay could serve as a beneficial alternative molecular approach for veterinarians in the clinical detection of feline infectious peritonitis, offering a simpler and potentially more accessible diagnostic option.

## 5. Conclusions

In conclusion, this study lays the groundwork for developing a colorimetric RT-LAMP-XO procedure for detecting FCoV in cats. The specially designed LAMP primers based nucleocapsid gene exhibit high specificity for FCoV. Compared to qPCR, the colorimetric RT-LAMP-XO method offers notable advantages in terms of convenience, and ease of visualization. Consequently, this RT-LAMP-XO method shows great promise as a diagnostic tool and could be utilized alongside other molecular approaches for addressing ongoing pandemic diseases in both animals and humans.

## Figures and Tables

**Table 1:** Oligonucleotide primers were used in this study. Degenerated bases were used according to IUPAC nomenclature: R (A/G), W (A/T), Y (C/T), and S (G/C).

Primer Name	Sequence (5′ → 3′)	Ref.
NF3	GCAGGYAARGGWGATGTG	This study
NB3	GCATCATCYTTTGGCAGG	
NFIP (NF1c-NF2)	GTAGCRTTTGGCAGCGWTAYGG-TGCTAGAWGTRSTTCAGCYAA	
NBIP(NB1c-NB2)	GTTCCATCAGTGTCTAGCRTG-ACTTTCACYTGRTCWCCARC	
NLF	RGYAACGAGATCACTATCACC	
NLB	GSYAGTCAATGGTCTGCTG	
F9N	CGTCAACTGGGGAGATGAAC	[28]
R9N	CATCTCAACCTGTGTGTCATC	

**Table 2:** Comparison of RT-LAMP and qPCR assays for FCoV detection.

	qPCR+	qPCR−	Total
RT-LAMP-XO +	17	0	17
RT-LAMP-XO −	0	60	60
Total	17	60	
(%) Sensitivity (95% CI)	100 (80.49% to 100%)		
(%) Specificity (95% CI)	100 (94.04% to 100%)		
FCoV prevalence	22.08 (13.42% to 32.98%)		
(%) Positive Predictive Value (95% CI)	100 (80.49% to 100%)		
(%) Negative Predictive Value (95% CI)	100 (94.04% to 100%)		
(%) Accuracy (95% CI)	100 (95.32% to 100%)		

## Data Availability

The data presented in this study are available within the article. Raw data supporting this study are available from the corresponding author.

## Supplementary Materials

The following supporting information can be downloaded at: https://www.mdpi.com/article/10.3390/v17030418/s1, Figure S1 In silico analysis of developed LAMP primer test with CCoV (KP981644)., Figure S2 In silico analysis of developed LAMP primer test with TGEV (NC_038861)., and Table S1: Clinical samples tested with RT-LAMP*-XO* and qPCR.
