# Supplementary material for: Colorimetric Reverse Transcription Loop-Mediated Isothermal Amplification with Xylenol Orange Targeting Nucleocapsid Gene for Detection of Feline Coronavirus Infection
Source: Viruses. 2025 Mar 14;17(3):418. doi: 10.3390/v17030418 (PMC11946810; doi:10.3390/v17030418)
Supplement: Supplementary file 1 [file viruses-17-00418-s001.zip › viruses-3476777-supplementary.pdf]

Supplementary Material

Colorimetric Reverse Transcription Loop-Mediated Isothermal Amplification with Xylenol Orange Targeting Nucleocapsid Gene for Detection of Feline Coronavirus Infection

Kotchaporn Khumtong [1, 2, §], Witsanu Rapichai [2, 3, §], Wichayet Saejung [1, 2], Piyamat Khamsingnok [2], Nianrawan Meecharoen [4], Siriluk Ratanabunyong [3], Hieu Van Dong [2, 5], Supansa Tuanthap [6], Amonpun Rattanasrisomporn [7], Kiattawee Choowongkomon [3], Oumaporn Rungsuriyawiboon [8] and Jatuporn Rattanasrisomporn [1, 2,*]

[1] Graduate Program in Animal Health and Biomedical Sciences, Faculty of Veterinary Medicine, Kasetsart University, Bangkok 10900, Thailand; kotchaporn.khu@ku.th (K.K.); wichayet.sa@ku.th (W.S.), fvetjpn@ku.ac.th (J.R.)
[2] Department of Companion Animal Clinical Sciences, Faculty of Veterinary Medicine, Kasetsart University, Bangkok 10900, Thailand; tswitsanu@gmail.com (W.R.); piyamat.kha@ku.th (P.K.)
[3] Department of Biochemistry, Faculty of Science, Kasetsart University, Bangkok, 10900, Thailand; ae.med@hotmail.com (S.R.); fsciktc@ku.ac.th (K.C.)
[4] Central Laboratory (CTL), Center for Veterinary Research and Innovation, Faculty of Veterinary Medicine, Kasetsart University; Bangkok 10900, Thailand; nianrawan.mee@ku.ac.th (N.M.)
[5] Faculty of Veterinary Medicine, Vietnam National University of Agriculture, Hanoi 131000, Vietnam; dvhieuvet@vnua.edu.vn (D.V.H.)
[6] Faculty of Veterinary Medicine, Rajamangala University of Technology Tawan-ok, Bangpra, Chonburi 20110, Thailand; supansa_tu@rmutto.ac.th (S.T.)
[7] Interdisciplinary of Genetic Engineering and Bioinformatics, Graduate School, Kasetsart University, Bangkok 10900, Thailand; fgraapr@ku.ac.th (A.R.)
[8] Department of Veterinary Technology, Faculty of Veterinary Technology, Kasetsart University, Bangkok, 10900 Thailand; cvtopr@ku.ac.th (O.R.)
These authors contributed equally to this work.
* Correspondence: fvetjpn@ku.ac.th (J.R.)

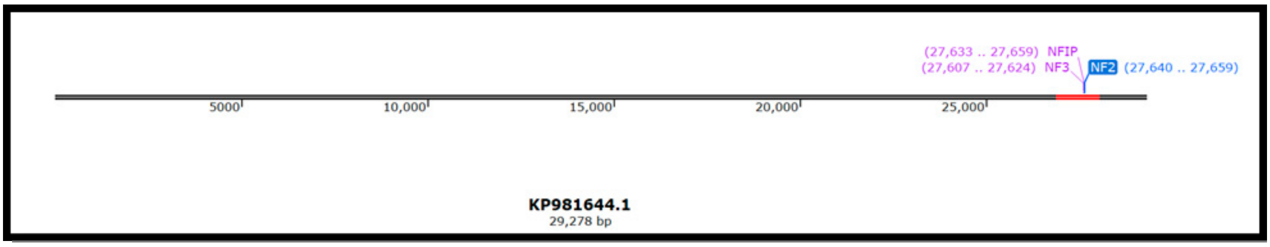

Figure S1 *In silico* analysis of developed LAMP primer test with CCoV (KP981644). Red colour strip is N gene region. NF3, NFIP and NF2 are our developed LAMP primer showing partial specificity of primer set.

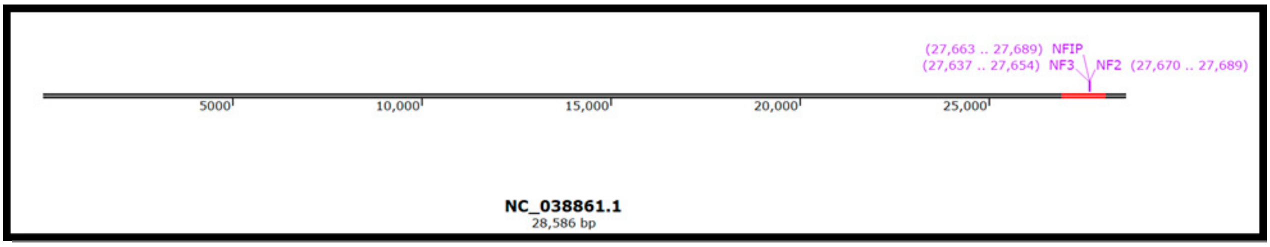

Figure S2 *In silico* analysis of developed LAMP primer test with TGEV (NC_038861). Red colour strip is *N* gene region. NF3, NFIP and NF2 are our developed LAMP primer showing partial specificity of primer set.

Table S1. Clinical samples tested with RT-LAMP-XO and qPCR

Name	RT-LAMP-XO	qPCR (Ct value)
**KU82**	Negative	Negative
**KU83**	Positive	Positive (27.42)
**KU84**	Negative	Negative
**KU85**	Negative	Negative
**KU86**	Positive	Positive (27.83)
**KU87**	Negative	Negative
**KU88**	Negative	Negative
**KU89**	Negative	Negative
**KU90**	Negative	Negative
**KU91**	Negative	Negative
**KU92**	Negative	Negative
**KU93**	Negative	Negative
**KU94**	Negative	Negative
**KU95**	Negative	Negative
**KU96**	Positive	Positive (23.25)
**KU97**	Negative	Negative
**KU98**	Negative	Negative
**KU99**	Negative	Negative
**KU100**	Negative	Negative
**KU101**	Negative	Negative
**KU102**	Negative	Negative
**KU103**	Negative	Negative
**KU104**	Negative	Negative
**KU105**	Positive	Positive (20.00)
**KU106**	Negative	Negative
**KU107**	Negative	Negative
**KU108**	Negative	Negative
**KU109**	Negative	Negative
**KU110**	Negative	Negative
**KU111**	Positive	Positive (24.66)
**KU112**	Negative	Negative
**KU113**	Negative	Negative
**KU114**	Negative	Negative
**KU115**	Negative	Negative
**KU116**	Negative	Negative
**KU117**	Negative	Negative
**KU118**	Positive	Positive (25.00)

**KU119**	Negative	Negative
**KU120**	Negative	Negative
**KU121**	Positive	Positive (22.21)
**KU122**	Negative	Negative
**KU123**	Positive	Positive (22.54)
**KU124**	Negative	Negative
**KU125**	Negative	Negative
**KU126**	Negative	Negative
**KU127**	Negative	Negative
**KU128**	Negative	Negative
**KU129**	Negative	Negative
**KU130**	Negative	Negative
**KU131**	Negative	Negative
**KU132**	Negative	Negative
**KU133**	Negative	Negative
**KU134**	Negative	Negative
**KU135**	Positive	Positive (24.35)
**KU136**	Positive	Positive (22.44)
**KU137**	Negative	Negative
**KU138**	Negative	Negative
**KU139**	Negative	Negative
**KU140**	Positive	Positive (26.54)
**KU141**	Negative	Negative
**KU142**	Negative	Negative
**KU143**	Negative	Negative
**KU144**	Negative	Negative
**KU145**	Positive	Positive (25.33)
**KU146**	Positive	Positive (25.20)
**KU147**	Positive	Positive (24.32)
**KU148**	Positive	Positive (25.60)
**KU149**	Negative	Negative
**KU150**	Negative	Negative
**KU151**	Negative	Negative
**KU152**	Negative	Negative
**KU153**	Negative	Negative
**KU154**	Positive	Positive (23.09)
**KU155**	Negative	Negative
**KU156**	Negative	Negative

**KU157**	Positive	Positive (25.39)
**KU158**	Negative	Negative
